# Antibody-mediated cell depletion therapies in multiple sclerosis

**DOI:** 10.3389/fimmu.2022.953649

**Published:** 2022-09-12

**Authors:** Alice Mariottini, Paolo A. Muraro, Jan D. Lünemann

**Affiliations:** ^1^ Department of Brain Sciences, Imperial College London, London, United Kingdom; ^2^ Department of Neurosciences, Drug and Child Health, University of Florence, Florence, Italy; ^3^ Department of Neurology with Institute of Translational Neurology, University Hospital Münster, Münster, Germany

**Keywords:** antibody, therapy, multiple scleorsis (MS), immunotherapy, immune reconstitution

## Abstract

Development of disease-modifying therapies including monoclonal antibody (mAb)-based therapeutics for the treatment of multiple sclerosis (MS) has been extremely successful over the past decades. Most of the mAb-based therapies approved for MS deplete immune cell subsets and act through activation of cellular Fc-gamma receptors expressed by cytotoxic lymphocytes and phagocytes, resulting in antibody-dependent cellular cytotoxicity or by initiation of complement-mediated cytotoxicity. The therapeutic goal is to eliminate pathogenic immune cell components and to potentially foster the reconstitution of a new and healthy immune system. Ab-mediated immune cell depletion therapies include the CD52-targeting mAb alemtuzumab, CD20-specific therapeutics, and new Ab-based treatments which are currently being developed and tested in clinical trials. Here, we review recent developments in effector mechanisms and clinical applications of Ab-based cell depletion therapies, compare their immunological and clinical effects with the prototypic immune reconstitution treatment strategy, autologous hematopoietic stem cell transplantation, and discuss their potential to restore immunological tolerance and to achieve durable remission in people with MS.

Multiple sclerosis (MS) is the most common chronic inflammatory, demyelinating disease of the central nervous system (CNS), afflicting more than 2.5 million people worldwide **(** 1 **)**. MS develops in young adults with a complex predisposing genetic trait and probably requires inciting environmental insults to trigger the disease. Both experimental and clinical evidence suggest that MS is an autoimmune disease with dysregulated adaptive immunity at its core. Genome-wide association studies have revealed multiple associations with immune-system-related gene variants, most importantly the HLA-DR15 haplotype (2); focal MS lesions are thought to be caused by the infiltration of immune cells, including T cells, B cells and myeloid cells, into CNS parenchyma (3); a substantial fraction of T and B cells isolated from CNS lesional tissue and the cerebrospinal fluid (CSF) from MS patients are derived from clonal expansion (4, 5); intrathecally produced oligoclonal antibodies present in the CSF show evidence for antigen-dependent affinity maturation (6); various immunotherapies targeting lymphocyte survival, function or migration show beneficial treatment effects (7); and some facets of MS can be mimicked in experimental animal models largely driven by autoimmune T cells and collectively termed experimental autoimmune encephalomyelitis (EAE) (8). Long considered a variant of MS, Neuromyelitis optica (NMO) is a separate, rare disease entity characterized, in most cases, by selective but not exclusive involvement of the optic nerve and spinal cord, also evolving with a relapsing-remitting clinical course. Pathogenic immunoglobulin G (IgG) antibodies binding to aquaporin-4 (AQP4), a water channel located at the terminal feet of astrocytes in the blood–brain barrier, are detectable with high specificity in patients with NMO, but not in MS. The term NMO spectrum disorders (NMOSD) was coined to include rare CNS syndromes with predominant optic nerve and spinal cord involvement even in absence of AQP4-specific IgG. While patients with NMOSD benefit from immunosuppressive therapy, not all treatment modalities effective in MS are equally effective in NMOSD, indicating that underlying disease mechanisms are different (9).

Although there is still no curative treatment for (MS), disease-modifying medications have developed enormously during the last 30 years. Interferon-β and glatiramer acetate were the first modalities approved for the therapy of MS three decades ago. The introduction of oral medications including sphingosine 1-phosphate receptor modulators, fumarates, dihydroorotate dehydrogenase inhibitors, and purine nucleoside analogues was a second important milestone (10), followed by the development of recombinantly produced monoclonal antibodies (mAbs) for MS therapy. Natalizumab, a recombinant humanised anti-α4-integrin antibody marketed as Tysabri^®^, was the first mAb to be approved in the USA and Europe for the treatment of MS in 2004 (11). Natalizumab blocks the interaction of α4 integrins with their ligands, thereby inhibiting migration and trafficking of leukocytes through the blood-brain barrier and the extracellular matrix within the central nervious system. Most of the mAbs currently approved for the treatment of MS employ a different, immunosuppressive mechanism of action: they deplete pathogenic immune cells through pro-inflammatory Fc receptor-mediated effector mechanisms. In addition to immune cell-depletion, some of these therapies might re-establish immune tolerance through short-term immunosuppression followed by rebuilding of a new and healthy immune system. This review illustrates recent developments in the design and effector mechanisms of Ab-based cell deletion therapies in MS, evaluates clinical benefits and risks of currently approved treatment modalities and provides an outlook on emerging and future developments. To examine immune reconstitution effects as well as measures of efficacy, we compare Ab-based therapies with autologous haematopoietic stem cell transplantation (AHSCT), a treatment strategy involving mobilization and storage of haematopoietic stem/progenitor cells and intensive immune ablation followed by re-infusion of the autologous stem/progenitor cells and repopulation of the lympho- and haematopoietic systems (12).

## Effector mechanisms of cell-depleting therapeutic Abs in MS

**Figure 1:**
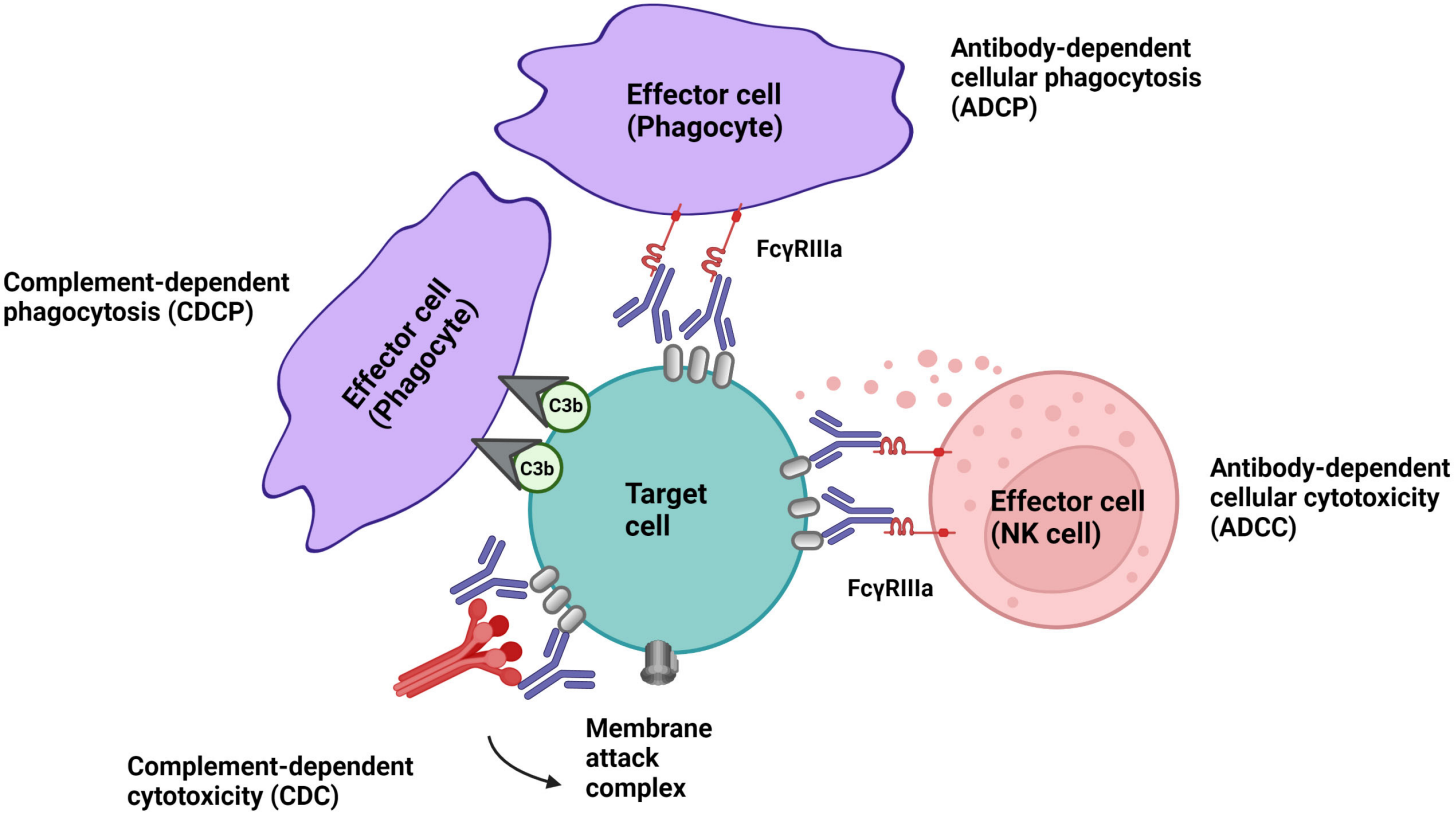
Effector mechanism of cell-depleting therapeutic IgG antibodies in MS. Complement-dependent cytotoxicity (CDC) is triggered through binding of C1q, which initiates activation of the classical complement pathway. Signalling through activating Fc receptors (FcγRs) expressed by innate immune cells such as natural killer cells and phagocytes initiates antibody-dependent cellular cytotoxicity (ADCC) and antibody-dependent phagocytosis (ADCP). Membrane-bound complement cleavage products such as C3b can additionally function as opsonins by interacting with complement receptors on effector cells which can result in complement-dependent cellular phagocytosis (CDCP).

Cell-depleting therapeutic IgG antibodies in MS lyse target cells through at least three mechanisms (**Figure 1**): I.) antibody-dependent cellular cytotoxicity (ADCC) triggered by signaling through activating Fc receptors (FcγRs) expressed by natural killer cells; II.) complement-dependent cytotoxicity (CDC) through binding of C1q, which initiates activation of the classical complement pathway; and III.) antibody-dependent cellular phagocytosis (ADCP) mediated by phagocytes recognizing opsonized target cells. Membrane-bound complement cleavage products such as C3b or C4b also function as opsonins by interacting with complement receptors on effector cells which can result in complement-dependent cellular phagocytosis (CDCP) (**Figure 1**).

Immune cell-depleting Abs currently approved for MS therapy include alemtuzumab and CD20-targeting Abs. Alemtuzumab is a humanized monoclonal IgG1 kappa antibody that selectively targets CD52, an antigen highly expressed on T and B cells, to a lesser extent on innate immune cells, but not on haematopoietic stem cells (HSCs) (13). It is currently approved by the FDA and EMA for highly active relapsing‐remitting (RR) MS, and data from initial treatment cohorts as well as extension studies of phase III clinical trials provided evidence that alemtuzumab can induce prolonged disease remission with no evidence for disease activity in more than 50% of treated patients (14, 15).

CD20^+^ B cell-depleting therapy is highly effective against relapsing forms of the disease, and is also the first treatment approach proven to prevent disability worsening in primary progressive MS (PP-MS). Rituximab, initially developed and approved for the therapy of B cell-lymphomas, is effective in the treatment of several autoimmune diseases including MS (16). Ocrelizumab (Ocrevus ^®^), a second-generation humanized anti-CD20 antibody, is approved by the FDA, the EMA and other regulatory agencies for the treatment of relapsing MS and PP-MS. Ofatumumab (Kesimpta ^®^) is a fully humanized CD20-specifc Ab approved by the FDA and EMA for the treatment of active relapse-onset MS.

**Figure 2:**
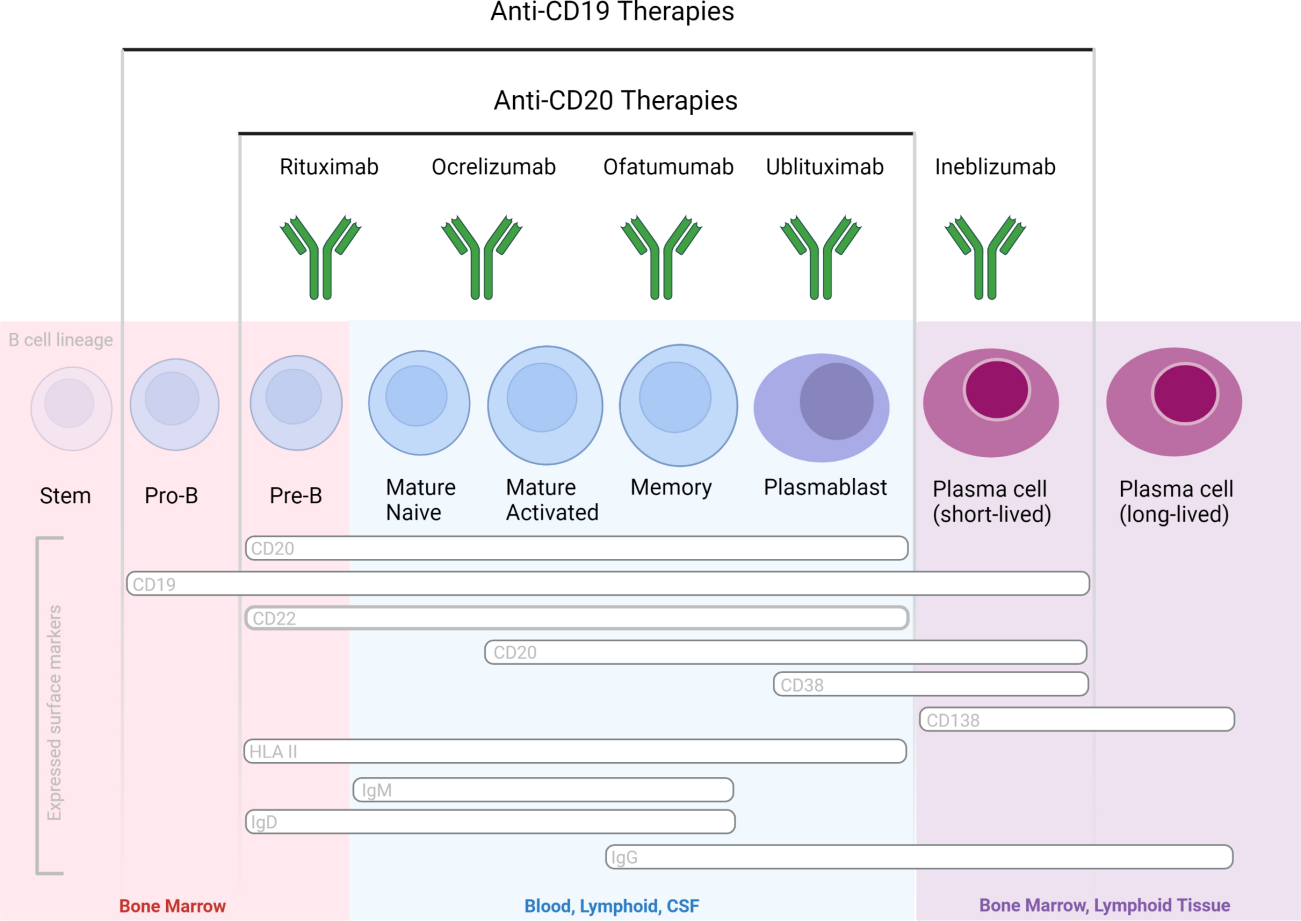
B cell-depleting mAbs with proven efficacy in MS and NMOSD, based on phase 3 randomized controlled clinical trials. Therapeutic Abs differ in their molecular design and targeted epitopes.

All of the three aforementioned Abs are of the human IgG1 subclass and target the CD20 antigen, expressed by a broad range (immature, transitional, naïve and memory) of B cells but not by plasma blasts/cells, and by approximately 5% of circulating T cells (17). Therapeutic anti-CD20 mAbs differ, however, from each other in their structure, epitopes targeted within the CD20 molecule, binding affinity to cell-surface CD20, and their mechanisms of action of B cell depletion (**Figure 2**). Depending on their capacity to cluster CD20 on the cell surface, anti-CD20 antibodies are grouped into type I and type II, the latter not having this ability (18, 19). Type I antibodies like rituximab, ocrelizumab and ofatumumab have been shown to efficiently activate complement, presumably because clustering facilitates the formation of hexameric IgG-Fc platforms suitable for C1q binding (20, 21). While both type I and type II antibodies are able to induce ADCC as well as ADCP (19), type II antibodies appear to be superior in inducing direct cell death (22, 23). *In vitro* data indicates that rituximab depletes through both FcγRs as well as complement dependent effector mechanisms. Ocrelizumab, equipped with humanized Ab backbone, exhibits greater ADCC compared to CDC than rituximab, but also depletes B cells through antibody-dependent cellular phagocytosis. Ofatumumab, a fully human monoclonal antibody was designed for greater CDC than ADCC activity (24–27) and is currently the only anti-CD20 mAb administered in a subcutaneous, rather than intravenous, dosing regimen. To what extent these different effector mechanisms contribute to cell-depleting or, in general, therapeutic Ab activity *in vivo* is less well understood and might depend on the disease condition treated and on the organ environment in which the antibody mediates its activity (28). It has become clear across many pre-clinical animal model systems that cytotoxic antibody binding to cellular FcγRs is critical for their therapeutic activity *in vivo* (29, 30).

Ab effector functions are regulated by a single biantennary glycan of the heavy chain, which resides just below the hinge region, and the presence of specific sugar moieties on the glycan has profound implications on IgG effector functions. The majority of circulating IgG antibodies are fucosylated which, compared to afucosylated isotypes, reduces IgG´s binding affinity for the activating FcγRIII (CD16) and thereby its potential to induce ADCC (31, 32). Fucosylation also appears to impair ADCP (33, 34). Consequently, afucosylation of therapeutic cell-depleting mAbs results in improved target cell elimination.

Ublituximab is a novel type I chimeric, (IgG1) anti-CD20 mAb glycoengineered with a low fucose content in its fragment crystallizable (Fc) region to enhance affinity for all variants of FcγRIIIa receptors, thereby producing potent ADCC (35). Ublituximab targets a unique epitope on CD20 not targeted by other anti-CD20 mAbs and exhibits 100 times greater natural killer cell–mediated ADCC *in vitro* than rituximab in cells from patient donors with chronic lymphocytic leukemia (CLL) (36). The potent ADCC activity allow for administering lower doses and shorter infusions times versus other presently available anti-CD20s mAbs. Two identical Phase 3, randomized, multi-center, double-blinded, active-controlled studies, ULTIMATE-I (NCT03277261) and ULTIMATE-II (NCT03277248), are reported to show clinical efficacy in patients with relapsing forms of MS (37).

Inebilizumab is an afucosylated type 1 humanized IgG1 mAb specific for CD19 and depletes a wide range of B lineage cells, including plasmablasts and some plasma cells (38). Targeting of the latter two populations could have a therapeutic merit particularly in antibody-mediated neurological disease such as NMOSD. Efficacy and safety of inebilizumab in individuals with (NMOSD) and detection of pathogenic antibodies specific for aquaporin 4 were demonstrated in the randomized, double-blind, phase 3 placebo-controlled study (ClinicalTrials.gov identifier: NCT02200770) (Clinicaltrials.gov) (39). Due to its efficacy for targeting a broader range of B cells, including Ab-secreting cells unrecognized by anti-CD20 agents, inebilizumab may have a greater therapeutic impact than the aforementioned CD20-specific agents. The aforementioned RCTs reported an acceptable safety profile of inebilizumab in neurological patients. However, longer-term safety profiles of CD19-immunotherapies in neurological patients remain to be determined, specifically related to IgG depletion and risks for infectious diseases. Inebilizumab is currently undergoing clinical evaluation for kidney transplant desensitization, myasthenia gravis, and IgG4-related disease. Safety and tolerability of inebilizumab in MS has been tested in a phase 1 trial (40), but the clinical program to develop the drug for MS has been discontinued. (**Table 1**)

**Table 1:** Abbreviations used in the manuscript.

Ab: antibody AHSCT: autologous haematopoietic stem cell transplantation ARR: annualized relapse rate AQP4: aquaporin 4 Breg: regulatory B cells CDI: confirmed disability improvement CDI-FS: confirmed disability improvement-free survival CDW: confirmed disability worsening CDW-FS: confirmed disability worsening-free survival CI: confidence interval CNS: central nervous system CSF: Cerebrospinal fluid DMT: disease-modifying treatment EDSS: Expanded disability status scale IFN: interferon beta Ig: immunoglobulin mAb: monoclonal antibody MRI: magnetic resonance imaging NEDA: no evidence of clinical and radiological disease activity, defined as the absence of all the following: relapses, disability worsening and signs of inflammatory activity (new T2 lesions and/or gadolinium-enhancing lesions) at brain magnetic resonance imaging NEDA-S: NEDA survival, i.e. proportion of cases who had not yet experienced any clinical or radiological disease activity OLE: open-label extension phase of a randomized clinical trial OR: odds ratio PP-MS: primary-progressive multiple sclerosis R-FS: relapse-free survival R-MS: relapsing multiple sclerosis RR-MS: relapsing-remitting multiple sclerosis RCT: randomized clinical trial SD: standard deviation SP-MS: secondary-progressive multiple sclerosis Th: T helper cell Treg: regulatory T cells

## Effect of cell depleting-Abs on adaptive immune signatures in MS

Immunological studies on cell-depleting Abs, as well as those on AHSCT, provided important insights into mechanisms responsible for their clinical efficacy and safety profiles. We can distinguish for both on- and off-target (or unanticipated) effects, desirable and unwanted side effects (**Table 2**).

**Table 2:** Summary of on- and off-target effects on the immune system of therapeutic cell-depleting mAbs and relationship with benefits and risks of adverse events.

	On-target beneficial effect	On-target detrimental effect	Off-target (or unanticipated)beneficial effect	Off-target (or unanticipated)detrimental effect
**Alemtuzumab**	Ablation of disease-mediating B and T cells (41–43)	Prolonged T cell depletion, particularly CD4+ (42, 44)	Increase in regulatory T and B cell phenotypes (45–50) Changes in cytokine patterns: reduced production of pro-inflammatory (e.g. IL-6, IL-17, IL-21, IL-22, IFN- γ) and increase of anti-inflammatory cytokines (e.g. IL-7, IL-4, IL-10, TGF-β) (43, 45, 46, 50, 51)	Early B cell hyper-population (increased risk of antibody-mediated secondary autoimmunity) (45, 52–55) T cell repopulation driven by homeostatic proliferation (increased risk of T cell-mediated secondary autoimmunity) (51, 56)
**CD-20 depleting mAbs:** **Ocrelizumab** Ofatumumab* **Rituximab** Ublituximab*	Ablation of CD20+ B cells contributing with pleiotropic functions to disease pathogenesis (e.g. antigen presentation and activation of pathogenetic T cell clones) (16, 57–62). Ablation of potentially disease-mediating CD20+ T cells (5, 63–66)	Reduced humoral immunity (e.g. response to vaccination, increased risk of infections) (16, 57–59, 67–71)	Changes in B cell subsets after immune repopulation: skewing towards naïve and regulatory phenotypes (5, 65, 72) Modifications in the pattern of cytokine secretion (e.g. increase in IL-10; reduction in IL-6 and lymphotoxin) (65, 72–74) Reduced percentage and activation/proliferation of pro-inflammatory T cell phenotypes (Th17 and Th1) (64–66, 73, 75)	
**Inebilizumab**	Depletion of putative disease-mediating CD19+ B cell clones (40, 76)	Reduced humoral immunity (40, 76)		
**AHSCT**	Ablation of disease-mediating T cells (77–79) Ablation of B cells contributing to disease pathogenesis (79)	Transient reduction in cell mediated and innate immunity (increased risk of infections) (79–81). Transient reduction in myeloid-derived blood cells (79–81).	Immune cell reconstitution promoted by thymic reactivation with regeneration of T cell receptor repertoire diversity (80, 82, 83) Increase in regulatory function of B and T cells (79, 84–86) Reduced percentage and activation/proliferation of pro-inflammatory T cell phenotypes (Th17, Th1, MAIT) (79, 86–88) Reduced production of pro-inflammatory cytokines, with shift towards anti-inflammatory profile (77, 84, 86, 87, 89) Down-regulation of pro-inflammatory genes in adaptive and innate immunity cells (90–92)	Secondary autoimmunity (93)

*Most of the evidence on the effects of Ab-mediated cell depletion therapies were provided by studies on ocrelizumab and rituximab.

### Alemtuzumab

Alemtuzumab induces the reduction of circulating lymphocyte counts with little effect on monocytes, NK cells and granulocytes, promoting a rapid (within 1 month) and marked depletion of B (up to 85%) and T lymphocytes (up to 95% for CD4+ and 85% for CD8+ T cells), with similar dynamics following each course of treatment (42). After depletion, immune cell repopulation occurs with different rates across lymphocytes subsets.The median time to recover the lower limit of normal was shorter for B cells (7.1 months) compared with T cells (median of 20 and 35 months for CD8+ and CD4+ T cells, respectively), and values after 1 year of treatment were still reduced by 70% for CD4+ cells and by 50% for CD8+ cells (94, 95) Furthermore, over a median follow-up of 12 years (range 0.5 – 16) the recovery to baseline levels for CD8+ and CD4+ counts was reported in only 30% and 21% of the patients, respectively (44).

Alemtuzumab depletes both naïve and memory phenotypes, and the subsequent repopulation occurs according to differential patterns for T and B cells, inducing changes in the relative proportions of subsets compared to pre-treatment. In the T cell compartment, the immune cell repopulation is mainly driven by homeostatic proliferation of residual clones, inducing a relative decrease in naïve and increase in effector memory cells, especially in the T CD8+ pool, with a predominance of terminally differentiated memory CD45RA+ (TEMRA) cells for at least 24 months (45, 51). Consistent with the sustained peripheral proliferation of T cells, CD4 and CD8 Ki67 expression was increased for up to 2 years after alemtuzumab, as were markers of increased activation (HLA-DR) and susceptibility to Fas-mediated apoptosis (CD95), with evidence in both CD4+ and CD8+ cells of chronic activation and repeated cell division (51). CD4 expression of programmed death-1 (PD-1) and lymphocyte activation gene-3 (LAG-3) were increased 6–9 months after treatment (51). The lack of a relevant contribution of thymic reactivation is suggested by: (i) a reduced number of cells recently generated from the thymus identified by T cell receptor (TCR) excision circles (TRECs); (ii) the observation that up to month 12 following treatment T cells exhibit reduced clonality in the TCR repertoire, being the baseline high-frequency clones expanded post-treatment (51). Similar findings were reported also within the regulatory T (Treg) cell compartment (48).

As for B cells, a rapid and early hyper-repopulation is observed, achieving absolute values above baseline by 35.3% in CARE-MS I and by 26.8% in CARE-MS II (42), being higher increments (up to 165% of the baseline count) also reported (96). Opposite to the T cell pool, the repopulated B cell pool is dominated by immature phenotypes associated with an increase in B-Cell Activating Factor (BAFF) levels (96) The depletion of total memory B cells is sustained up to month 36 compared with baseline, and it is associated with a significant elevation of total naïve B cells from month 5 to 48, up to a 180% increase compared with pre-treatment levels (42, 49). Plasma cells express little or no CD52 and seem not to be affected by alemtuzumab (97, 98).

As an on-target effect, the lymphocyte depletion induced by alemtuzumab promotes a long-term abrogation of MS disease activity, which is thought to be mediated, at least in part, by the ablation of disease-mediating T and B cells. Compared with untreated MS patients and healthy controls, an increase in proliferative responses of T cells to self-antigens was observed following alemtuzumab treatment (41). However, this was associated with a marked increase in T cell apoptosis persisting for at least 18 months, with increased expression of caspase-3 in T cells and monocytes compared with untreated MS (41). More recently, the antigen-specific immune response to different CNS self-antigens and control non-self-antigens was evaluated in five patients by ELISpot, showing that both the cumulative number of IFN-γ secreting cells and the ratio of IFN-γ secreting cells/CD4+ T cell counts were reduced following treatment compared to baseline (43). Although Th1 counts were not assessed, the Authors speculated that these data might suggest that alemtuzumab induces functional changes in the immune cell repertoire.

Correlations between the kinetics of immune cell repopulation and disease activity were not consistently detected across studies, suggesting that subtle qualitative changes in immune cell subsets might be relevant to the therapeutic effects (99–101). Aligned with this observation, CD4+CD25+CD127+foxP3-Teff cells were recently suggested as a possible marker for monitoring disease activity, as patients with stable disease showed a trend for reduced levels of such population, with reduced Teff: Treg ratios (49). Furthermore, exploratory analyses using a random generalized estimating equation Poisson model suggested that the relative balance of several key potentially pathogenic and regulatory subsets might be associated with the risk for gadolinium-enhancing lesions, with an association between the risk for T2 lesions and CD3+CD8+CXCR3+ T cells (49).

A modification in the balance between cell subsets and in patterns of cytokine secretion following immune cell repopulation may therefore be considered as an unexpected off-target beneficial effect of alemtuzumab that may contribute to the abrogation of disease activity.

An increase in regulatory populations was described following alemtuzumab treatment, both in the T and B cell compartments (49). Briefly, an expansion in Treg compared with baseline was observed starting at month 1, with a more modest although significant elevation up to year 2 (45, 46). Treg predominantly exhibited a mature/activated phenotype and performed an effective suppression of induced proliferation of patients’ peripheral blood mononuclear cells at months 5 and 17 of treatment compared with CD25-depleted peripheral blood mononuclear cells with effective reduction in the IL-17 and IFN-γ responses upon myelin-basic protein (MBP) stimulation (49). These observations suggest that Tregs are functional and may exhibit enhanced suppressive activity in the early phase of repopulation after each course of alemtuzumab treatment (49). In another study, the suppressive function of post-depletion Treg from MS patients remained constantly increased compared to baseline over 24 months after alemtuzumab start, nearly reaching levels comparable to those seen in Treg obtained from healthy donors (48). The restoration of deficient counts of B cells expressing a “regulatory” phenotype (CD19+CD24^hi^CD38^hi^ and CD19+PD-L1^hi^) in the peripheral blood of relapsing MS patients was observed during the first year following alemtuzumab (47) Values of the naïve B “regulatory” phenotype CD19+CD20+CD27-CD24^hi^CD38^hi^ were significantly increased compared with baseline up to month 36 following treatment in another study, with a marked decrease in the ratio of total memory B cells to naïve “regulatory” cells (49). In a cross-sectional study, B cells collected from patients who had received alemtuzumab secreted significantly higher levels of IL-10 and brain-derived neurotrophic factor (BDNF) up to month 24 compared to a cohort of pre-treated MS patients (50). Furthermore, B cells from post-treatment cohorts showed a greater inhibition of the proliferation of autologous CD4+CD25- T cells as compared to the pre-treated cohort, suggesting enhanced regulatory ability of B cells up to month 24 after treatment commencement.

Changes in the relative proportion of CD4+ and CD8+ T cell subsets were demonstrated by the observation of an incremental increase in the percentage of TGF- β1, and IL-10–producing CD4+ and CD8+ T cells within 12 months from treatment, as well as of IL-4–producing Th2 and CD8+ T cells, whereas the percentages of IL-17A–producing Th17 cells and IFN-γ–producing Th1 and CD8+ T cells were significantly decreased (45, 51). A transient increase in the percentage of CD4+IFN-γ+ Th1 cells at month 5 compared with baseline was observed in another study (49). Consistent with Treg and Th2 expansion, gradually increasing serum levels of IL-7 and IL-4 were observed over the first 6 months after treatment compared with baseline, whereas cytokines produced by or polarizing towards Th17 and Th1 phenotypes (IL-17A, IL-17F, IL-21, IL-22, and IFN- γ; IL-11, IL-1β, IL-6, and IL-23, IL-27 and IL-12) were reduced (45)A significant reduction in concentrations of IL-2, IFN-γ and IL-17A compared to baseline was reported up to month 23 after treatment (49). Accordingly, mRNA levels of the anti-inflammatory cytokines IL-10, IL-27, and TGF-β significantly increased after alemtuzumab treatment compared with baseline (46). On the other hand, levels of proinflammatory cytokines and transcriptional factors related to the Th17 and Th1 subset, and of proinflammatory chemokines or chemokine receptors (CCR3, CCR4, CCR5, CCR6, CXCR3, CXCL10, CCL20, VLA4) significantly decreased. These modifications occurred during the first 12 months, and remained at values similar to month 12 over the subsequent year of follow-up (46). After alemtuzumab treatment, an increase of CD4+ and CD8+ T cells that express CXCR3 and CCR5 and of CD8+ T cells that express CDR3 and CCR4 was reported the majority of these cells expressed VLA-4, suggesting heightened trafficking potential in activated T cells (49).

Few data are available on the impact on the CSF compartment, and CSF oligoclonal bands (i.e. two or more oligoclonal IgG bands detected by separation of CSF proteins while not demonstrable in corresponding serum) (102) were still present in all the 15 cases tested before and after 3 to 28 months following alemtuzumab administration in one study (44). More recently, a decreased intrathecal IgG production was reported in association with reduced peripheral blood IgG levels, and oligoclonal bands disappeared in two cases at month 24 of follow-up (103).

Increased risk of infections might be considered as an unwanted on-target effect, that may be attributed at least in part to prolonged T cell depletion, particularly CD4+ (42). A progressive reduction in the concentrations of all (Ig)subgroups, that was described over 36 months after initiation of alemtuzumab therapy in one study, might also contribute to this adverse event, as reduced IgG concentrations were associated with an increased incidence of pneumonia, otitis, and sinusitis (103).

Secondary autoimmunity may be considered as an unexpected off-target detrimental effect of alemtuzumab, promoted by “unbalances” between cell subsets that take place during immune cell repopulation Both antibody-mediated and T-cell mediated autoimmune diseases have been described following alemtuzumab treatment (56).

Rapid hyper-population of B cells, which might not be adequately counterbalanced by Tregs (absolute number of CD4 Tregs reduced by 81% in CARE-MS I and 86.3% in CARE-MS compared with baseline) (45, 52), and homeostatic proliferation of T cells are supposed to contribute to this category of adverse events. Patients developing autoimmunity showed, compared to those who did not, reduced thymopoiesis at month 12 (51), and higher rates of T cell cycling, with increased proliferation and apoptosis but without differences in lymphopenia between groups (41) Occurrence of autoimmunity was associated with increased production of IL-21 in one study, possibly driven by a genetical susceptibility (41). Furthermore, a greater T cell clonal restriction following alemtuzumab treatment by both sequencing (increased high-frequency clones) and TCR CDR-3 length spectratyping (more undetectable BV families and reduced complexity of the remaining families) was associated with the development of autoimmunity (51). Serial TCR sequencing showed that T-cell pool remained oligoclonal at month 12 in one patient who developed autoimmunity, whereas TCR diversity had recovered by month 6 and was higher than pre-treatment at month 12 in another who did not (51). B cell hyper-population was suggested to drive also B cell-mediated tumefactive CNS demyelination reported in a few cases after alemtuzumab treatment (53–55) This event was associated with a decrease in B regulatory cells with CD19+CD24^hi^CD38^hi^ phenotype at the time of disease reactivation in one case (104).

### Ocrelizumab, ofatumumab and rituximab

Ocrelizumab induced a reduction of CD19+ cells to negligible levels by week 2 from treatment, whereas CD4+ T cells remained stable throughout the treatment period in the pivotal trials (59, 60). An initial 2–6% mean decrease from baseline in peripheral-blood counts of CD3+ or CD8+ cells was observed at week 2, followed by an additional 2-6% decrease for CD8+ cells from week 2 to the end of the trials (week 96 or 120) (59, 60).

In the phase 2 dose-finding trial MIRROR, ofatumumab (from 3 to 60-mg doses every 12 weeks, or 60 mg every 4 weeks) induced a dose-dependent depletion of B cells, being greater for the 60-mg dose every 4 weeks (to <2% of baseline levels at maximum depletion) and the 30- and 60-mg dose every 12 weeks (to ≈5% of baseline) than for the 3-mg dose every 12 weeks (to ≈25% of baseline) (61). Similar rates of B-cell repopulation were observed across groups, being B-cell repletion achieved by 64% to 74% of patients by week 24 after the last administration, but repopulation was slower for the higher-dose groups. After the loading dose of 60 mg, ofatumumab leads to B-cell depletion in the peripheral blood, which is sustained following subsequently monthly 20 mg dosing (69). By day 14 (i.e. after having received 40 mg-ofatumumab), B-cell counts were below the lower limit of normal in all the treated patients, with a median depletion by 99.1% of baseline values. A bioequivalent rapid B-cell depletion was observed with ofatumumab 20 mg every 4 weeks self-administered subcutaneously *via* autoinjector compared with the pre-filled syringe, independent of body weight quartiles (105). In ofatumumab-treated patients, a significant relationship between weighted mean B-cell count and new gadolinium-enhancing lesions was reported by *post-hoc* analyses, but moderate B-cell depletion (with circulating B-cell levels to ≈25% of baseline) was surprisingly effective in significantly reducing new gadolinium-enhancing lesions (relative reduction: 71%) (61). Partially explaining this finding, the observation from animal models that subcutaneous administration of antibodies allows more direct access to the lymph nodes compared with intravenous infusion, promoted by the absorption into the lymphatic system (106, 107). This may allow targeting B cells in the lymph nodes directly while sparing those in the spleen, a phenomenon that could help preserve the immunosurveillance (105).

Two doses of rituximab 1000 mg induced a rapid and near-complete depletion of CD19+ B lymphocytes (>95% reduction from baseline) starting from week 2 until week 24 and followed by gradual replenishment with a return to 31% of the baseline value by week 48 (16). Following two additional doses administered six months after the first course, one-third of the patients recovered B cell count at week 122 (57). Similar reductions compared with baseline were observed after subsequent courses of treatment, without a significant effect of the dose (< or ≥2 g each course) of rituximab administered, suggesting a possible ceiling effect (108) however, time to recovery of B cell count was faster for lower doses (250 mg) compared with higher doses (500 – 2000 mg) (109). Rituximab induced a similar pattern of depletion in NMOSD patients, followed by a repopulation promoted mainly by CD27- naïve B cells, that represented on average 86.2% of the B cell poo (58).

As an on-target effect, anti-CD20 mAbs promote the depletion of CD20 expressing cells, including subsets that may plausibly contribute to MS pathogenesis. Most of the CD20 bearing cells are represented by B cells, but the mechanisms underlying B cell role in disease pathogenesis are not fully understood: these might include pleiotropic functions involved in the interaction with the T cell compartment, such as cytokine secretion, antigen presentation, and driving of auto-proliferation of Th1 brain-homing CD4+ T cells by memory B cells (110). The beneficial effects of B cell depletion may be mediated, at least in part, by phenotypic and functional changes occurring in the re-populated B cell pool. This latter is characterized by a skewing towards naïve phenotypes after both ocrelizumab or rituximab administration, with an early decrease in naïve and memory B-cell numbers associated with an increase in percentages of plasmablasts and transitional B cells (5, 65). The subsequent replenishment is dominated by IgD+CD27- naïve and transitional B cell subsets, whereas memory B cell populations and IgD-CD27- B cells remained depleted for significantly longer, up to weeks 37–52 after rituximab administration (5, 65).

Modifications in the pattern of cytokine secretion towards an anti-inflammatory balance were also described, and they could contribute to reducing the state of activation and proliferation of pathogenetic T cells.

Relative to the total CD19+ B cell number, ocrelizumab induced a decrease in the percentage of B cells producing TNF-α, and an increase in the proportion of regulatory B cells producing IL-10 (65). However, the relative increase in the production of GM-CSF and frequency of IL-6 producing B cells suggest that complete restoration of immune tolerance was not achieved (65).

Opposite to this latter finding, repopulated B cells from MS patients treated with rituximab secreted reduced levels of IL-6, achieving values comparable with healthy controls (73). In another study, the secretion of IL-10 increased, whereas the frequencies of circulating GM-CSF+ B cells and secretion of GM-CSF decreased after treatment with rituximab (at a time when B cells were reconstituted to pre-treatment levels) compared to baseline, therefore resulting in an essentially normalized GM-CSF/IL-10 ratio (74). These changes were associated with decreased myeloid cell proinflammatory responses that persisted even after B cells reconstitution, in keeping with the diminished proinflammatory response profile of the reconstituted B cells. Similarly, rituximab promoted an enhancement of regulatory B cell function in NMOSD patients, as newly developed B cells secreted higher levels of IL-10 and decreased levels of lymphotoxin following depletion compared to baseline, consistent with the predominance of naïve B cells over memory phenotypes in the repopulated pool (72).

As an off-target unexpected effect plausibly mediated by enhanced regulatory function of B cells, modifications in T cell activation were also observed. A decrease in the percentages of CD4+ and CD8+ T cells producing IFN-γ to total CD4+ and CD8+ T cells, respectively, was reported following ocrelizumab treatment (65). Th1 (IFN-γ) and Th17 (IL-17) responses and proliferative responses of CD4+ and CD8+ T cells were reduced in MS patients treated with rituximab compared with baseline these modifications were suggested to be mediated by a reduced production of the pro-inflammatory cytokines lymphotoxin and TNF-α by the repopulated B cells (75).

In another study, IL-17 production in peripheral blood mononuclear cells from RR-MS patients treated with rituximab was lower than pre-treatment (without differences in IFN-γ), suggesting that this could be mediated by a reduced IL-6 production after B cell depletion (73).

Although the CD20+ pool is mostly constituted by B cells, CD20 is expressed also by a minor subset of T cells exhibiting an activated phenotype with increased production of proinflammatory cytokines (111). CD20+ T cells can be detected in peripheral blood, CSF and CNS lesions from MS patients and are considered possibly relevant to disease pathogenesis (112). The depletion of CD20+ T cells might therefore contribute to the therapeutic effectiveness of anti-CD20 mAbs, and its occurrence was independently reported following the administration of ocrelizumab or rituximab.

Two weeks after the first administration of 300 mg ocrelizumab, CD3+CD20+ T cells (representing at baseline almost 20% of the CD20+ cells) were rapidly and efficiently depleted from peripheral blood of MS patients (63) These findings were confirmed by another study showing a marked decrease in both absolute numbers and percentages of CD20+ T cells relative to CD3+ T cells six months after ocrelizumab start, being the relative reduction significant in both CD4+ and CD8+ subsets (65).

Similarly, a near-complete depletion of CD3+CD20+ T cells during weeks 1–12 after rituximab administration was reported, from a mean frequency of 7.8% to 0.36%, lasting for at least one year and followed by partial repletion (2.7%) starting from weeks 25–36 (5).

Mild and transient changes were observed after ocrelizumab administration also in the CD20- T cell population, with a decline in the proportion of effector T cells associated with a relative increase of CD8+ naïve T cells and the ratio of naïve/effector subsets for both CD4+ and CD8+ T cells, suggesting the redistribution of the T-cell compartment favoring naïve vs effector phenotypes (65).

Different effects on the T cell pool were observed after rituximab administration. No significant changes were reported in the whole CD3+ T cell counts in three studies on MS patients (5, 16, 57), whereas an early and transient reduction in CD3+ cells (by on average 25%) was observed in the first three months after treatment in a mixed cohort of MS and NMOSD patients, without significant changes compared to baseline following subsequent courses of treatment (108). Different factors, such as concomitant treatment, previous exposure to immunosuppressive drugs and presence of lymphopenia at baseline in a subset of cases, could contribute, at least in part, to this latter finding No significant changes in the numbers of CD4+, CD8+ cells nor in the CD4+/CD8+ ratio over long-term follow-up were observed in another study on MS and NMOSD patients (109).

Cell depletion was evident also in the CSF, where, at week 24 after 4 weekly doses of rituximab 375 mg/m^2^, the number of CD19+ B cells and CD3+ T cells were reduced by 90% and 55% compared with baseline, respectively (113). In the same study, no significant differences in IgG concentration, IgG index, IgG synthesis rate or number of oligoclonal bands were observed . A significant decline of CSF levels of CXCL13 (a chemoattractant factor for B cells and activated T cells) was reported following treatment with rituximab, with a positive correlation between CXCL13 and the degrees of reduction of T cell counts in the CSF (114). Based on these observations, the Authors speculated that B cells may indirectly promote reduced T cell trafficking across the blood-brain barrier involving the reduced CNS production of chemoattractant factors, such as CXCL13, by non-B cells.

As an on-target detrimental effect, serum Ig levels were reduced by CD-20 depleting mAbs, with potential implications for long-term safety.

At week 96 of the pivotal trials of ocrelizumab in R-MS, proportions of treated patients with Ig levels below the lower limit of normal were 1.5% and 2.4% for IgG and IgA, respectively, and 16.5% for IgM; no impact on existing antibody titers for mumps, rubella, varicella, or pneumococcus was reported (59). After five years of treatment, serum Ig levels were further reduced, being below the lower limit of normal in 5.4% and 5.1% of the cases for IgG and IgA, respectively, and in 29.5% for IgM, compared to 0.5% for IgG and IgM and 1.2% for IgG at baseline (70). Similar proportions were observed in ocrelizumab-treated PP-MS patients, being cases with IgM levels below the lower limit of normal 15.5% at week 120, compared with 1.2% of those in the placebo group (60). These proportions increased to 29% for IgM and 5% for both IgG and IgA at 6.5 study years of follow-up (71). In a pooled population of R-MS and PP-MS patients who received ocrelizumab in the pivotal trials, serious infections following episodes of a drop in Ig concentrations below the lower limit of normal were rare (67). Most of the infections (mainly represented by urinary tract infections, cellulitis, and pneumonia) resolved with standard treatment, without requiring ocrelizumab withdrawal.

Similar rates of patients with Ig deficiency were observed during ofatumumab treatment, being the proportion of cases with IgM levels below the lower limit of normal 14.3% (69).

A reduction of IgM levels below the lower limit of normal was reported in 22% and 32% of MS patients who had received 2 or 4 1000 mg-administrations of rituximab, respectively, with higher rates reported for longer exposures to treatment (16, 57), IgM were reduced in 37% of the NMOSD patients at year 5 of treatment, and IgA and IgG were reduced in 30% and 13% of the cases, respectively (58).

As a consequence of the impairment in antibody production, B cell-depleting therapies might interfere with the generation of an effective response to vaccination, as suggested by the observation that MS patients receiving ocrelizumab showed an attenuated humoral response compared to those receiving interferon or no treatment to clinically relevant non-live vaccines (tetanus toxoid, pneumococcal polysaccharide, pneumococcal conjugate vaccine, seasonal inactivated influenza) administered 12 weeks after treatment initiation (68). However, recent evidence suggests that cell-mediated immune response may preserve vaccine efficacy in B-cell depleted MS patients, although differential effects depending on the specific vaccination strategy adopted cannot be excluded (115, 116).

### Ublituximab

CD19+ B cells were efficiently depleted in most patients within 24 hours of receiving the initial 150 mg dose of ublituximab within 2 weeks after the second infusion, B-cell count was reduced by at least 95% from baseline, being the reduction sustained without significant recovery both at week 24 (pre-dose) and at week 48 of follow-up (62). Similarly, an immediate B cell depletion after a single 450 mg-dose of ublituximab was observed in five NMOSD patients treated in a pilot study, with a sustained depletion in B cell count ≤0.2% for two months in 4/5 cases, being 0.9% in the remaining one (117).

A transient but significant reduction in the percentage of T cells with respect to the total peripheral blood mononuclear cells count (from 43% of baseline to 29% of day 2) was observed about 24 hours after ublituximab administration, reverting to baseline by week 2. This phenomenon could be interpreted as a relative decrease of T cells in the peripheral blood due to efflux of myeloid cells from the bone marrow in response to the rapid depletion of B cells, as suggested by the observation, in the same timeframe, of a significant increase in the percentage of myeloid cells (64).

As for T cell subsets, a relative increase in the proportions of naïve CD4+ and CD8+ T cells with a reciprocal decrease in effector and central memory CD4+ and CD8+ T cells, without changes in the CD4+/CD8+ ratio, was observed during the first 24 weeks of ublituximab treatment (64). These modifications were significant starting from week 12 for the CD4+ T cells, whereas for CD8+ T cells the change in naïve/memory ratio occurred immediately on day 2, suggesting that some CD8+ T cells bearing the CD20+ marker may have been deleted by ublituximab. The depletion of CD20+ T cells by ublituximab was confirmed in MS patients by a subsequent study, showing an early (within 24 hours from the first administration) and long-term depletion of the vast majority of CD20+ T cells, being these latter naïve cells (as determined by CD45RA) in >95% of the cases (66). The modifications in the T cell subset persisted after the third ublituximab infusion performed at 24 weeks, up to week 48 (66).

A significant decline in the percentage of Th1, but not in IL-17+ and GM-CSF+ CD4+ T cells, and an increase in the percentage of Tregs (CD4+CD25^hi^Foxp3+ T cells) was observed over time in MS patients treated with ublituximab (64), and the proportion of Tregs doubled baseline values at week 48 (66). Furthermore, there was a significant decrease in the ratio of Th1:Tregs, Th17:Tregs, and CD4+GM-CSF+:Tregs, suggesting a shift in the T cell profile towards anti-inflammatory phenotypes, plausibly able to effectively regulate potentially pathogenic CD4+ T cells in MS (66).

### Inebilizumab

Inebilizumab treatment in NMOSD patients induced a robust depletion of CD20-positive B cells, that was maintained for at least 4 years (76). In a phase I study on 21 MS patients treated with different doses of inebilizumab, rapid B-cell depletion was observed at all dosing regimens except the 60-mg subcutaneously dose; the extent and duration of the B-cell depletion were dose-dependent, with most dose groups maintaining 90% depletion throughout the 24-week follow-up period (40). To our knowledge, no data are available so far on the effect of inebilizumab on T and B cell phenotypes or activation status in MS or NMOSD.

As an unwanted on-target effect, concentrations of IgG, IgM, IgA, and IgE decreased over time during treatment with inebilizumab. Most participants (76%) maintained normal IgG levels, and no significant associations were observed between the lowest reported IgG categories and severe infections, although the relatively small number of the participants could have prevented from finding significant correlations (76). In a phase I study on MS patients, total Ig levels at week 24 showed a mean percentage decrease of 10.5% from baseline, rising to 15.0% at the 18-month follow-up (40). The reduction was greatest for IgM, but was observed in all the Ig subtypes, although the total Ig levels remained within the normal range in all the study patients, and no reduction in the tetanus titer was observed.

### AHSCT

Substantial immunological changes, particularly affecting adaptive immunity, have been reported in patients undergoing AHSCT for autoimmune disorders, including major contributions from studies in people with MS. The topic of immune reconstitution following AHSCT in MS has been recently reviewed in detail (118). Among the most relevant effects that support the notion of “immune resetting” we highlight: the demonstration of thymic reactivation with regeneration of T cell receptor repertoire diversity (80); the demonstration of ablation of pre-treatment T cell repertoire in blood and CSF, and replacement with new T cells (83); and enhanced immune cell regulation (85). There is some evidence of regeneration of the B cell pool, with expansion of naïve B cell frequency after AHSCT (79), yet more research is needed to fill gaps in knowledge of the effects of AHSCT on the B cell compartment, which are potentially also relevant to the mechanism of action.

## Clinical efficacy of immune-cell depleting Abs in MS

Relapses, disability and signs of focal inflammation at brain MRI are used, alone or in combination, to assess the effectiveness of DMTs in MS.

Clinical outcomes focus on relapses and disability. Relapses are defined as acute or sub-acute episodes of new or increasing neurological dysfunction followed by full or partial recovery, in the absence of fever or infection (119). The impact of a DMT on relapses is usually estimated using the following outcome measures: relapse-free survival (R-FS), i.e. cumulative proportion of subjects who have not yet experienced a relapse at a definite timepoint (120); and/or annualized relapse rate (ARR), defined as the number of relapses that occur during a specific timeframe, adjusted to a one-year period.

Disability is measured with the expanded disability status scale (EDSS), a non-linear scale ranging from 0 (absence of neurological signs and symptoms) to 10 (death due to MS) (121). Disability milestones are EDSS scores of 4.0 (limited walking ability, being the patient able to walk without aid or rest for more than 500 m), 6.0 (need for unilateral aid to walk for about 100 m without rest) and 7.0 (essential restriction to wheelchair). Confirmed disability worsening (CDW) is usually defined as an increase of at least 1.0 or 0.5 points if baseline EDSS was <5.5 or ≥5.5, respectively (or of 1.5 points if baseline EDSS was 0), confirmed at a subsequent neurological evaluation performed after 12 or 24 weeks. Disability outcomes are usually expressed as CDW-free survival (CDW-FS), and/or EDSS changes over a definite period of observation. More recently, confirmed improvement of disability (CDI) has been included as disability outcome in clinical trials. CDI is usually defined as a confirmed decrease in EDSS score of at least 1.0 or 0.5 points if baseline EDSS was <5.5 or ≥5.5, respectively.

MRI inflammatory activity is defined as the presence at brain MRI of gadolinium-enhancing lesions and/or new or enlarging T2 focal lesions compared with a reference scan (122). More complex radiological measures, such as brain atrophy, are also evaluated in recent trials, but are not yet routinely adopted in clinical setting due to the complexity of post-processing analyses required for their estimation (123).

No evidence of disease activity (NEDA-3) is a combined clinical and radiological outcome measure referring to the absence of all the following: relapses, EDSS worsening and MRI activity (as defined above) (124).

**Figure 3:**
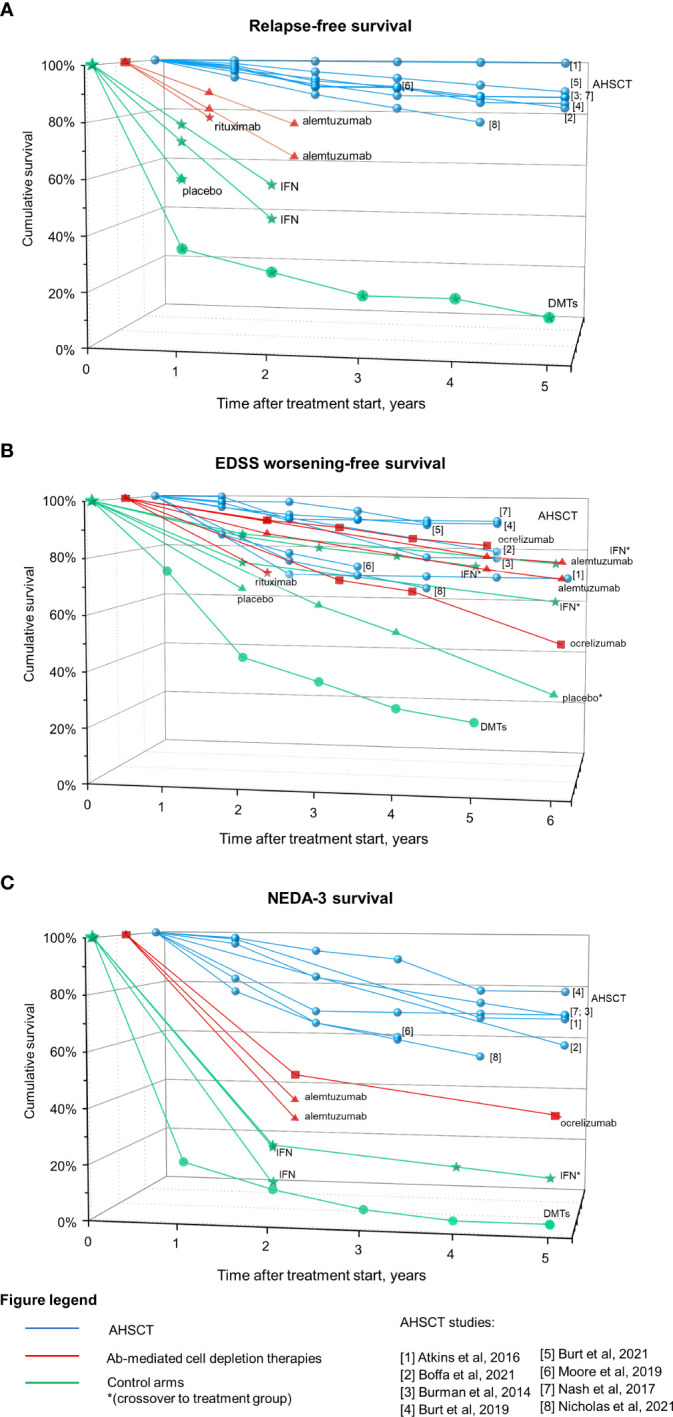
Efficacy on Ab-mediated cell depletion therapies and AHSCT on clinical and radiological measures of disease activity in MS. Data derived from AHSCT studies (blue), treatment arm of RCTs on Ab-mediated cell depletion therapies (red) and corresponding control arms (green) are reported as cumulative rates of survival free from relapses **(A)**, EDSS worsening **(B)**, and combined clinical-radiological disease activity (NEDA-3, **(C)**. Patients originally randomized to the control arm who were switched to the treatment under investigation during the OLE phase are marked with *. The control arm of the MIST trial on AHSCT is also depicted (DMTs), including patients who had failed DMTs prior to inclusion and received DMTs of higher efficacy or a different class than the therapy they were taking at the time of randomization. Differences in the characteristics of the patient populations included across trials (such as the selection of patients with highly active disease while on treatment and the lack of escalation in a subset of the cases for the DMTs group) could explain, at least in part, the fact that the DMTs group from the MIST trial performed lower than the placebo group of other trials.

**Figure 3** summarizes clinical efficacy of Ab-mediated cell depletion therapies and AHSCT on relapses (panel A), EDSS worsening (panel B) and clinical-radiological disease activity (NEDA-3; panel C) in patients affected by MS (data from randomized clinical trials -RCTs - and prospective randomized studies for Ab-mediated cell depletion therapies, and from prospective and retrospective cohort studies for AHSCT). The group DMTs represents the control arm of AHSCT in the MIST trial, and includes patients who received different treatments (interferons, glatiramer-acetate, dimethyl-fumarate, teriflunomide, fingolimod, natalizumab, rituximab, mitoxantrone, cyclophosphamide) “of higher efficacy or a different class than the therapy they were taking at the time of randomization” (125).

### Reducing MS relapses

#### Alemtuzumab

In two (RCTs) the efficacy and safety of alemtuzumab were compared with interferon beta 1a (IFN) in people with RR-MS who were naïve to treatments (CARE-MS I) or had relapsed while taking IFN or glatiramer acetate (CARE-MS II) (94, 95). In both the trials, the patients were randomized, in a 2:1 ratio, to receive alemtuzumab (12 mg per day) infused intravenously on 5 days at baseline and 3 days at 12 months, or IFN (44 μg) given subcutaneously three times per week after dose titration. CARE-MS II originally included an additional arm (alemtuzumab 24 mg) in which recruitment was prematurely closed after a protocol amendment, and the cases treated in this arm were excluded from primary analyses. Patients in the CARE-MS I were affected by early MS (inclusion criteria: naïve, disease duration up to 5 years and Expanded Disability Status Scale - EDSS - score up to 3.0), whereas those enrolled in the CARE-MS II were affected by a more advanced disease (inclusion criteria: disease duration up to 10 years and EDSS score up to 5.0). Occurrence of at least one relapse in the year before enrollment was required in both the studies. Primary analyses included 187 IFN-treated and 376 alemtuzumab-treated patients in the CARE-MS I, and 202 IFN-treated and 426 alemtuzumab-treated patients in the CARE-MS II trial. The mean EDSS score at baseline was 2.0 in CARE-MS I and 2.7 in CARE-MS II; the mean disease duration and age were 2.0 years and 33 years in CARE-MS I, 4.5 years and 35 years in CARE-MS II, respectively. At year 2, R-FS was significantly higher in the alemtuzumab group compared with the IFN group in both the trials (p<0.0001). For alemtuzumab-treated patients, R-FS was 77.6% (95% confidence interval, CI, 72.9 – 81.6) in CARE-MS I and 65.4% (95% CI 60.7 – 69.7) in CARE-MS II, compared with 58.7% (95% CI 51.1 – 65.5) and 46.7% (95% CI 39.5 – 53.5) for the IFN-treated patients in the two trials, respectively. Alemtuzumab reduced the risk of relapses compared to IFN by 54.9% in CARE-MS I and by 49.4% in CARE-MS II, being the yearly relapse rate on alemtuzumab treatment of 0.18 (95% CI 0.13 – 0.23) and 0.26 (95% CI 0.21 – 0.33), respectively.

Most of the alemtuzumab treated patients from the CARE-MS I and II trials were enrolled in a 4-year open-label extension (OLE) study. The results were provided as 3-year interim analyses for alemtuzumab treated patients in the core studies (defined as alemtuzumab-only group) (15, 126), and as a final 6-year report including also the outcomes of originally IFN-treated patients who switched to alemtuzumab at enrollment in the OLE (defined as IFN-alemtuzumab group) (127). During the OLE, alemtuzumab re-treatment due to disease activity (up to six courses in 0.3 – 1% of the cases) was received by 35.5% and 45.7% of MS patients enrolled in the core studies CARE-MS I and II, respectively treatment with other DMTs was administered in 2.6% of the patients from CARE-MS I and in 10.1% of those from CARE-MS II (127).

(ARR)remained low during the OLE, with a cumulative ARR over years 0-6 of 0.16 and 0.24 for the alemtuzumab-only groups from the core CARE-MS I and II, respectively (127). The proportion of MS patients free from relapses annually over years 3-6 was 84-89% in naïve (from CARE-MS I) and 79-87% in previously treated patients (from CARE-MS II) . As expected, a significant reduction in ARR compared to the core study was observed in the IFN-alemtuzumab groups, with a cumulative ARR in years 3-6 of 0.12 and 0.15 for patients from CARE-MS I and II, respectively.

#### Ocrelizumab

In two identical RCTs (OPERA I and OPERA II), people affected by relapsing MS (R-MS; PP-MS excluded) with active disease (defined as the presence of relapses over the previous 2 years) were randomized, in a 1:1 ratio, to receive intravenous ocrelizumab at a dose of 600 mg every 24 weeks or subcutaneous IFN at a dose of 44 μg three times weekly for 96 weeks (59). MS patients included in the analyses were 827 and 829 for the ocrelizumab and IFN arms, respectively. The mean age at enrolment was 37 years; the mean EDSS score at baseline was 2.8, and patients were naïve to treatment in most of the cases (71 – 75% across groups). The ARR on ocrelizumab treatment was 0.16 compared with 0.29 for IFN-treated patients, with a 46% and 47% lower rate with ocrelizumab in the OPERA I and II trials, respectively (p<0.001).

More than 94% of MS patients who completed the core OPERA I and II trials were enrolled in an OLE phase of 3 years, during which patients previously treated with ocrelizumab continued the treatment, whereas those treated with IFN switched to ocrelizumab 600 mg every 24 weeks (70). Through years 1-5, ARR remained low in MS patients receiving continuous ocrelizumab (ranging from 0.07 in year 5 to 0.14 over year 1), whereas in previously IFN-treated patients it was significantly reduced by 52% after switching to ocrelizumab (ARR 0.10 in year 3, and 0.07 in year 5). The difference in ARR was not significant between the two groups over years 3 to 5 (p=0.8, 0.97, and 0.7, respectively).

#### Ofatumumab

In the two identical RCTs ASCLEPIOS I and II, 1882 patients with active R-MS (PP-MS excluded) were randomized, in a 1:1 ratio, to receive ofatumumab at a dose of 20 mg subcutaneously every 4 weeks (after 20-mg loading doses at days 1, 7, and 14), or oral teriflunomide at a dose of 14 mg once daily, for up to 30 months (69). Patients were affected mostly by RR-MS (94% of the cases), with a mean age of 38 years and baseline EDSS of 3.0; on average, and 38-40% of the cases were naïve to treatment. Over a median follow-up of 1.6 years, ARR was 0.11 (95% CI 0.09 – 0.14) and 0.10 (95% CI 0.08 – 0.13) in the ofatumumab groups compared with 0.22 (95% CI 0.18 – 0.26) and 0.25 (95% CI 0.21 – 0.30) in the teriflunomide groups The relapse rate ratio was 0.49 (95% CI 0.37 – 0.65; p<0.001) and 0.42 (95% CI 0.31 – 0.56; p<0.001) for the ASCLEPIOS I and II trials, respectively. Analyzing pooled data from the two trials, R-FS at year 2 was 82.3% in ofatumumab-treated vs 69.2% in teriflunomide treated patients (128).

Long-term data on the safety (primary objectives) and effectiveness (secondary objectives) of ofatumumab will be provided by the ongoing open-label umbrella OLE study ALITHIOS, that enrolled 1701 R-MS patients who had previously participated in ofatumumab trials (phase 3 ASCLEPIOS, and phase 2 APOLITOS and APLIOS) (129).

#### Rituximab

In the phase 2 RCT HERMES, 104 patients affected by active RR-MS (i.e. with relapses over the previous year) were randomized in a 2:1 ratio to receive intravenous rituximab (1000 mg) or placebo on days 1 and 15, and followed up to week 48 (16). Mean age at inclusion was 39.6 (standard deviation - SD - 8.7) years in the rituximab and 41.5 (SD 8.5) years in the placebo arm, and median EDSS was 2.5 (range 0 -5) in both the arms; on average, 22% of the cases were naïve to treatment. The proportion of cases without gadolinium-enhancing lesions at baseline was unbalanced between groups, being 63.8% and 85.7% in the rituximab and placebo arms, respectively (p=0.02). The proportion of patients with relapses was reduced in the rituximab group compared with the placebo group both at weeks 24 (14.5% vs. 34.3%, p= 0.02) and 48 (20.3% vs. 40.0%, p=0.04), with an increased risk of relapse with placebo of 2.3 (90% CI 1.3 – 4.3) at week 24, and 1.9 (90% CI 1.1 - 3.2) at week 48. ARR was lower in the rituximab group compared with the placebo group at 24 weeks (0.37 vs. 0.84, p=0.04), but not at 48 weeks (0.37 vs. 0.72, p=0.08).

The efficacy of rituximab in PP-MS was explored in the phase 2/3 RCT OLYMPUS, where 439 people with PP-MS were randomized 2:1 to receive two doses (two weeks apart) of intravenous rituximab 1000 mg or placebo every 24 weeks through 96 weeks, for a total of 4 courses (57). The presence of active disease (i.e. relapses or MRI inflammatory activity) was not required as an inclusion criterion. The mean age at enrolment was 49.9 years (SD 8.9), the median EDSS score was 5.0 (range 2.0 – 6.5); 64.9% of the cases were naïve to treatment and gadolinium-enhancing lesions at baseline were observed in 24.5% of the cases. During the trial, relapses were observed in 2% of the patients in the rituximab group and 3.4% of those in the placebo group.

In an open label phase II trial, switching to rituximab from first line injectable therapies in clinically stable RR-MS patients induced a reduction in the number of gadolinium-enhancing lesion and in mean levels of CSF neurofilament light chains compared to the 3-months the run-in period (130), whereas another open label study randomizing 73 SP-MS patients to rituximab or glatiramer-acetate showed no differences between groups in relapse activity, nor in EDSS worsening (131).

The beneficial and adverse effects of rituximab as ‘first choice’ and as ‘switching’ therapy for adults with MS were recently explored in a Cochrane systematic review including 5 RCTs and 10 controlled non‐randomized studies of interventions comparing rituximab with placebo or approved DMTs (132). Overall, 6,429 participants (13,143 affected by R-MS, and 3286 with progressive MS) were included; over one to two years study duration, rituximab was compared as ‘first choice’ with placebo (1 RCT) or other DMTs (1 non‐randomized study of intervention ), or as ‘switching’ against placebo (2 RCTs) or other DMTs (2 RCTs, 9 non‐randomized studies of interventions. According to the Authors’ conclusion, rituximab as ‘first choice’ and as ‘switching’ therapy may compare favourably with a wide range of approved DMTs for preventing relapses in R-MS, whereas its protective effect against disability worsening was uncertain. For progressive MS, the effect of rituximab could not be determined due to the limited information available.

#### Ublituximab

In a phase 2 study, 48 patient with active R-MS (i.e. with relapses in the previous 2 years) were randomized, in a 3:1 ratio, to receive intravenous ublituximab or placebo over a study period of 48 weeks (62). The administration of the drug was performed on days 1 (initial dose 150 mg), and 15, and then at week 24 (maintenance dose 450 or 600 mg). Patients were enrolled in six cohorts with different doses and rates of infusion, and those allocated in the placebo arm crossed over to receive ublituximab at day 28. The primary endpoint of the study was the proportion of ublituximab-treated patients with at least 95% peripheral CD19+ B cell depletion from baseline at week 4, whereas clinical and radiological outcomes of response to treatment were investigated as secondary or exploratory endpoints. The mean age at inclusion was 40 years (SD 10), mean EDSS was 2.44 (SD 1.36); 33% of the patients were naïve to treatment. R-FS (secondary endpoint) at week 48 was 93%, and the ARR on treatment (0.07) was reduced by 95% compared to the year prior to study entry (1.45).

#### Inebilizumab

In a phase 2/3 RCT trial (N-MOmentum), 230 people affected by (NMOSD) were randomized, in a 3:1 ratio, to receive intravenous inebilizumab (300 mg) or placebo on days 1 and 15 (38). After a randomized controlled period of 197 days, patients were offered participation in an OLE phase, where participants who were treated with inebilizumab during the core study received an additional dose of inebilizumab (300 mg on day 1), and those treated with placebo received two doses of inebilizumab (300 mg each) 15 days apart; subsequently, 300 mg inebilizumab was administered every 26 weeks. Enrolment in the N-MOmentum trial was prematurely halted in 2018 because of a clear demonstration of efficacy. History of NMOSD attack requiring rescue therapy in the previous two years was required for inclusion; at enrolment, patients had on average 43 years and median EDSS was 3.5 (range 0 – 8.0) in the inebilizumab arm and 4.0 (range 1 – 8.0) in the placebo arm; 32-34% of the cases had not received any previous maintenance therapy. (AQP4) antibodies were positive in 92% - 93% of the cases. R-FS was higher in the inebilizumab group compared to the placebo group, being at day 197 86.7% and 59.9%, respectively (hazard ratio, HR 0.27, 95% CI 0.15 – 0.50; p<0.0001), and the treatment effect was similar in the AQP4-positive subgroup. At week 52 of the OLE, R-FS was 84% in the group receiving continuative treatment with inebilizumab and 51% in the placebo-inebilizumab group (133), and interim analyses of a subgroup of 75 AQP4-positive participants who had received inebilizumab for at least 4 years show an R-FS of 83%, and stabilization of the mean EDSS score in the overall cohort (76).

### Inhibiting disability progression

#### Alemtuzumab

In treatment naïve RR-MS patients (CARE-MS I trial), 8.0% (95% CI 5.7 – 11.2) of MS patients in the alemtuzumab group showed 24-week (CDW) at year 2, being the difference not significant compared with the IFN arm (11.1%, 95% CI 7.3 – 16.7, p=0.22); accordingly, changes in EDSS score from baseline did not differ between the alemtuzumab and IFN groups (p=0.97) (94). At year 6 of the OLE, differences in disability outcomes were not significant, and (CDW-FS) compared with the core study baseline was 78% for the alemtuzumab-only group and 80% for the IFN-alemtuzumab group (127).

In previously treated RR-MS patients (CARE-MS II trial), the proportion of cases with CDW at year 2 was 12.7% (95% CI 9.9 – 16.3) in the alemtuzumab group, with a significant risk reduction of 42% compared with IFN (proportion with CDW: 21.1%, 95% CI 15.9 - 27.7, p=0.0084) (95 EDSS change from baseline was different between the two groups and EDSS score improved of mean -0.17 points in the alemtuzumab arm, whereas it worsened of mean 0.24 points in the IFN arm (p<0.0001). At year 6 of the OLE, CDW-FS was 72% in the alemtuzumab-only group and 67% in the IFN-alemtuzumab group, being the difference not significant (127).

At year 2 of the CARE-MS II trial, (CDI) was observed in 28.8% (95% CI 24.2 – 34.1) of the cases in the alemtuzumab group (mean change: -0.17 EDSS points) compared to 12.9% (95% CI 8.3 – 19.3) of those in the IFN group (mean change: +0.24 EDSS points), p=0.0002 (95). At year 6 of the OLE, EDSS improvement was detected in 24% of MS patients in the alemtuzumab-only group, and in 16% of those in the IFN-alemtuzumab group from CARE-MS II, being the cumulative proportion of patients with CDI significantly higher at each year from year 1 through year 6 (127). On the other hand, no significant differences were observed in CDI between the alemtuzumab-only and the IFN-alemtuzumab groups from the core CARE-MS I trial (21% vs 24%, respectively) (127).

#### Ocrelizumab

In the OPERA I and II trials, the cumulative proportion of patients with 24-week CDW at week 96 was 7.6% in MS patients treated with ocrelizumab compared to 12% in those receiving IFN, with HR of 0.60 (95% CI 0.43 – 0.84; p=0.003) (59). During the OLE, the proportion of patients with 24-week CDW from baseline remained significantly lower in MS patients receiving continuous ocrelizumab compared with those switching from IFN to ocrelizumab at the end of the core trials, being 16.1% vs 21.3% at the end of year 5 (p=0.014), with an HR for CDW during the OLE phase (i.e. with rebaselining at start of the OLE) of 1.06 (95% CI 0.8–1.41; p = 0.7) (Hauser et al., 2020).

Twelve-week CDI was reported in 20.7% and 15.6% of the pooled populations from OPERA I and II who received ocrelizumab and IFN, respectively, with a 33% higher rate of improvement with ocrelizumab, that was significant in the OPERA I trial only (p=0.02) (59). During the OLE, the proportion of MS patients with 24-week CDI was numerically higher in the continuous ocrelizumab-treated patients compared to switchers, although the difference reached significance only at year 5 (25.8% vs 20.6%; p = 0.046) (70). The HR for improvement was not significant in both the overall study period and the OLE only (i.e. after rebaselining at the start of the OLE period), being 1.31 (95% CI 0.96–1.78; p=0.06) and 0.89 (95% CI 0.61–1.31; p=0.6), respectively .

The effectiveness of ocrelizumab in PP-MS was explored in the RCT ORATORIO, where 732 PP-MS were randomized in a 2:1 ratio to receive intravenous ocrelizumab (600 mg) or placebo every 24 weeks for at least 120 weeks (60). The median age at inclusion was 46 years (range 18 – 56), and the median EDSS was 4.5 (range 2.5 – 7.0). Over a median trial duration of 2.9 years in the ocrelizumab group and 2.8 years in the placebo group, the proportion of patients with 24-week CDW was 29.6% and 35.7% in the ocrelizumab and placebo groups, respectively (HR 0.75; 95% CI 0.58 – 0.98, p=0.04; relative risk reduction: 25%). Ninety-five per cent of the participants who concluded the ORATORIO trial entered, after an extended controlled treatment period, the OLE phase where patients randomized to ocrelizumab during the core study continued to receive it (i.e. the continuous ocrelizumab group) and those who were randomly assigned to the placebo group were switched to ocrelizumab 600 mg every 24 weeks at the start of the OLE phase (i.e. the placebo to ocrelizumab group, equivalent to a delayed start cohort); interim results over at least 6.5 study years (48 weeks/year) of follow-up were provided as *post-hoc* analyses (71). The proportion of PP-MS patients showing 24-week CDW was lower in the continuous ocrelizumab group compared with the placebo to ocrelizumab group, being 51.7% vs 64.8% (difference 13.1%, 95% CI 4.9 – 21.3; p=0.0018) at 6.5 study years of follow-up. Patients in the continuous ocrelizumab group had an HR for CDW of 0.72 (95% CI 0.58 – 0.89; p=0.0021) compared with the delayed start cohort, but there were no significant differences in EDSS accrual between the two groups when using the start of the OLE as baseline.

#### Ofatumumab

At year 2 in the ASCLEPIOS I and II trials, the cumulative proportion of cases with 24-week CDW was 8.1% in ofatumumab-treated patients compared with 12. 0% in teriflunomide-treated cases, with an HR of 0.68 (95% CI 0.50 to 0.92; p=0.01) (69). At the same timepoint, proportions of patients with CDI were similar between the two groups, being 11.0% in MS patients receiving ofatumumab and 8.1% in those receiving teriflunomide (HR 1.35; 95% CI 0.95 - 1.92; p=0.09).

#### Rituximab

In the OLYMPUS trial, the cumulative proportion of patients with 12-week CDW (primary endpoint) was 20.2% at week 48 and 30.2% at week 96 in rituximab-treated PP-MS, being similar to that observed in the placebo group (19.3% and 38.5% at the two time points, respectively, p=0.144) (57). Similarly, at week 96, the proportion of patients with 24-week CDW did not differ between the two arms (exploratory endpoint: 27.3% in the rituximab and 30.4% in the placebo group, p=0.59), nor did the mean EDSS change between baseline and last follow-up (0.33 and 0.45 in the rituximab and placebo arms, respectively, p=0.34). However, rituximab tended to delay the time to CDW compared with placebo, with HR of 0.77. Planned subgroup analyses of the primary endpoint according to baseline characteristics showed that age and presence of gadolinium-enhancing lesions were predictors for treatment effect: rituximab delayed time to CDW compared with placebo in treated patients aged <51 years (HR 0.52; p=0.010) or those with gadolinium-enhancing lesions at baseline MRI (HR 0.41; p=0.007); the presence of both these characteristics showed an additive predictive effect (HR 0.33, 95% CI 0.14 – 0.79; p=0.009).

#### Ublituximab

In a phase 2 study on patient with R-MS treated with ublituximab, 24-week CDW-FS (exploratory endpoint) was 92% at week 48, whereas 17% of the patients showed CDI at the same timepoint (62).

#### Inebilizumab

In the N-MOmentum trial, not confirmed EDSS score worsening from baseline at the last visit was observed in 16% of NMOSD patients treated with inebilizumab compared with 34% of those in the placebo arm, with OR 0.37 (95% CI 0.18 – 0.74; p=0.0049) (38). Prespecified subgroup analyses showed a consistently reduced risk of EDSS worsening in inebilizumab-treated participants compared with placebo regardless of the baseline EDSS score, number of previous attacks, or disease duration. Furthermore, *post-hoc* analyses confirmed a lower proportion of 12-week CDW in the inebilizumab group compared with the placebo group, being of 5.7% and 14.3%, respectively (HR 0.37; 95% CI 0.15 – 0.95; p=0.039) (134).

In the first year of the OLE, where all the patients received inebilizumab, the mean change in EDSS decreased in both originally placebo- and inebilizumab- treated cases (133).

### Inhibiting MRI activity

#### Alemtuzumab

MRI inflammatory activity (new or enlarging T2 lesions and gadolinium-enhancing lesions) was lower in the alemtuzumab group compared to the IFN group in both the CARE-MS trials (94, 95). At year 2, the proportion of alemtuzumab-treated RR-MS patients who were free from new or enlarging T2 lesions ranged from 52% to 54% (vs 32 - 42% of IFN treated patients), being 91 to 93% of the patients free from gadolinium-enhancing lesions (vs 77 - 81% of IFN treated patients).

Cumulative MRI inflammatory activity-free survival (MRI-FS) over years 3-5 of the OLE was 53.8% and 48.6% in alemtuzumab-only patients from the CARE-MS I and II core trials, respectively (15, 126).

At year 6 of the OLE, MRI-FS was 66% - 70% in the alemtuzumab-only groups, and 67% - 71% in the IFN-alemtuzumab groups a significant reduction in MRI activity was observed in the IFN-alemtuzumab groups starting from year 3 of the OLE compared with the core studies (127).

#### Ocrelizumab

Ocrelizumab reduced the number of new or enlarging T2 lesions by 77% and 83% in the OPERA I and II trials, respectively (mean number of new or enlarging T2 lesions per T2-weighted MRI scan: 0.32 and 0.33 in the ocrelizumab groups compared with 1.41 and 1.90 in the IFN groups, p<0.001) (59). Most of the new or enlarging T2 lesions in the ocrelizumab groups were observed between baseline and week 24, with a reduction compared with IFN by 94-96% during the weeks 24 – 48, and by 97-98% during the weeks 48 to 96. The total mean number of gadolinium-enhancing lesions per T1-weighted MRI scan was 0.02 with ocrelizumab compared with 0.29 and 0.42 for IFN in the OPERA I and II trials, respectively, corresponding to a reduction in the ocrelizumab group by 94% and 95% (59). The proportion of R-MS patients without new or enlarging T2 lesions was 61.7% and 60.9% in the ocrelizumab groups, and 38.7% and 38% in the IFN groups (p<0.001), whereas the proportion of cases without gadolinium-enhancing lesions was 91.7% and 90.2% in the ocrelizumab groups and 69.8% and 63.9% in the IFN groups (p<0.001) (59).

In R-MS, continuous administration of ocrelizumab during the OLE maintained the near-complete suppression of MRI disease activity seen in the core phase, with an unadjusted rate of total T1 gadolinium-enhancing lesions and of new or newly enlarged T2 lesions of 0.006 and 0.031 over year 5, respectively (70). Switching from IFN to ocrelizumab induced an almost complete and sustained suppression of MRI lesion disease activity from year 3 to 5, MRI lesion counts were similar to those observed in continuous ocrelizumab-treated patients during the OLE, except for new T2 lesions at year 3 (higher number in switcher compared with continuous ocrelizumab-treated).

Similar results were observed in PP-MS during the OLE phase of the ORATORIO trial, with persistent suppression of MRI inflammatory activity in patients receiving continuous ocrelizumab, and almost complete and sustained suppression of new MRI lesion disease activity throughout the OLE in those switching from IFN to ocrelizumab. No differences were observed in T1 gadolinium-enhancing and new or enlarging T2 lesion counts between the two groups, except for new or enlarging T2 lesions at two timepoints where lesion numbers were already very low (71).

#### Ofatumumab

The mean number of gadolinium-enhancing lesions per T1-weighted MRI scan was 0.01 and 0.03 with ofatumumab compared to 0.45 and 0.51 with teriflunomide in ASCLEPIOS I and II, respectively, corresponding to a relative reduction with ofatumumab by 97% and 94%, respectively (69). The mean numbers of new or enlarging lesions per year on T2-weighted MRI scans were 0.72 and 0.64 with ofatumumab, compared with 4.00 and 4.15 with teriflunomide in the two trials, corresponding to a reduction with ofatumumab by 82% and 85% (p<0.001) in ASCLEPIOS I and II trials, respectively.

In a *post-hoc* analysis of pooled data from the two trials, survival free from gadolinium-enhancing lesion activity over two years was 54.1% in ofatumumab compared with 27.5% in teriflunomide treated patients (128).

#### Rituximab

In RR-MS patients from the HERMES trial, rituximab reduced by 91% total gadolinium-enhancing lesion counts compared with placebo at each study week analysed, beginning at week 12 (primary endpoint), with a mean of 0.5 and 5.5 gadolinium-enhancing lesions in the rituximab and placebo groups, respectively (p<0.001) (16). The proportion of cases without new gadolinium-enhancing lesions was 84.8% with rituximab and 54.3% with placebo (p<0.001). As for T2 lesions, the reduction in the volume of T2 lesion load from baseline to weeks 24 and 36 was greater in MS patients who received rituximab than in those who received placebo (p=0.008 and 0.004, respectively).

In the OLYMPUS trial, rituximab significantly reduced the median increase in T2 lesion volume compared with placebo from baseline to week 96, being 302 mm^3^ and 809 mm^3^ in the two groups, respectively (57).

#### Ublituximab

In R-MS, ublituximab suppressed the occurrence of gadolinium-enhancing lesions at weeks 24 and 48 of a phase 2 study (secondary endpoint), with a 100% reduction from baseline (mean number of gadolinium-enhancing lesions: 3.63, SD 7.80; p=0.003) (62). Occurrence of new or enlarging T2 lesions was observed in 15% of the cases between baseline and week 24, and in 2% of the cases between weeks 24 and 48, with a mean number of 0.20 (SD 0.43) and 0.04 (SD 0.29) new lesions, respectively. The MRI-FS was 83% at week 48.

#### Inebilizumab

In patients with NMOSD, inebilizumab significantly reduced compared with placebo the cumulative number of active MRI lesions (i.e. gadolinium-enhancing, or new or enlarging T2 lesions), being the mean number 1.6 (SD 1.0) and 2.3 (SD 1.3), respectively (relative ratio: 0.57, 95% CI 0.39 – 0.83) (38).

### Reaching status of “no evidence of clinical and radiological disease activity”

#### Alemtuzumab

At year 2, the proportion of RR-MS patients showing NEDA (NEDA survival, NEDA-S) was 39% and 32% in alemtuzumab-treated patients from the CARE-MS I and II trials, respectively, compared with 27% and 14% of the IFN treated patients from the same trials (94, 95). These differences corresponded to an odds ratio (OR) favouring alemtuzumab over IFN of 1.75 (95% CI 1.17 – 2.61) for the CARE-MS I and of 3.03 (95% CI 1.89 – 4.86) for the CARE-MS II trial

Cumulative NEDA-S over years 3-5 of the extension was 39.5% and 27% in alemtuzumab-only patients from the CARE-MS I and II core trials, respectively (15, 126).

#### Ocrelizumab

In R-MS patients treated with ocrelizumab, NEDA-S was 47.9% and 47.5% compared with 29.2% and 25.1% of IFN-treated patients for the two OPERA trials (p<0.001), but these findings were considered to be nonconfirmatory as a result of failure of the hierarchical analysis (59). Data from the OLE showed a higher proportion of patients with NEDA in the ocrelizumab compared with the IFN group during both the core OPERA I-II studies (at year 2: 48.5% vs 27.8%; p < 0.001) and the overall observation period (at year 5: 35.7% vs 19.0%; p < 0.001; relative increase with ocrelizumab: 88%); the difference between groups was significant also considering the OLE phase only (65.4% vs 55.1%; p<0.001) (70).

#### Ofatumumab

In a *post-hoc* analysis of pooled data from the ASCLEPIOS I-II trials, the proportion of R-MS patients achieving NEDA at month 12 was 47% in the ofatumumab group compared with 24.5% in the teriflunomide group (OR 3.36, 95% CI 2.67 - 4.21; p<0.001) (128). From month 12 to 24, proportions of patients with NEDA were 87.8% and 48.2% in the ofatumumab and teriflunomide groups, respectively (OR 8.09, 95% CI 6.26 - 10.45; p<0.001).

#### Ublituximab

At week 48, 74% of the R-MS patients treated with ublituximab achieved NEDA (exploratory endpoint) (62).

## Adverse events of Ab-mediated cell depletion therapies

*Administration-associated reactions* (most commonly headache, rash, pruritus, throat irritation nausea and pyrexia) were the most frequent adverse events in MS patients receiving Ab-mediated cell depletion therapies, and were reported in 90% of the alemtuzumab-treated patients (being serious in 3% of the cases) (94, 95), in 34 - 40% of ocrelizumab-treated patients (being mild to moderate in 98% of the cases) (59, 60), and 67-78% MS patients treated with rituximab (mild-moderate in 93% of the cases) (16, 57), with decreasing rate and severity over subsequent administrations. Systemic and injection-site associated reactions were reported in 20% and 11%, respectively, of R-MS patients treated with ofatumumab in the ASCLEPIOS I-II trials, mostly occurring at the first injection and being severe in 0.2% of the cases (69). Fifty per cent of R-MS patients receiving ublituximab experienced infusion-related adverse events, mostly on the first infusion day and all mild to moderate in severity (62). The incidence of infusion-associated reactions was similar in NMOSD patients receiving inebilizumab or placebo (9% and 11%, respectively) in the N-MOmentum trial (38).

*Infections* (most commonly upper respiratory, urinary tract and herpetic) were reported in 67% and 77% of the patients treated in the CARE-MS I and II trials, respectively, and were serious in 2-4% of the cases, but no life-threatening or fatal events were observed nor events requiring treatment discontinuation (94, 95). Incidence of infections peaked at year 1 after initiating treatment (60%), with a cumulative exposure-adjusted incidence rate of 8 and 10 events per 100 patients-years during years 0-2 and 3-6, respectively; serious infection incidence did not exceed 1.8% throughout the OLE up to year 6 (127).

Infections were observed in 57-60% of R-MS and 70% of PP-MS patients treated in the OPERA I-II and ORATORIO trials, respectively (59, 60). Upper respiratory and urinary tract infection and nasopharyngitis were the most common, with severe events in 1.3% of the RR-MS and 6% of the PP-MS treated patients; herpesvirus-associated infections were reported in 5-6% of the cases, mostly mild to moderate in severity. During the OLE of the OPERA trials, the rate of infections in R-MS per 100 patient-years was 75, and serious events occurred at a rate of 1.5 per 100 patient-years over the 5-year period (70). In PP-MS patients from the ORATORIO OLE, the rate of infections per 100 patient-years was 73, consistent with the rate observed in both the ocrelizumab-treated and placebo groups from the core trial the rate of serious infection was 4.13 per 100 patient-years, similar to that of the placebo group in the core trial (71).

A reduction in serum Ig levels was observed over the OLE in ocrelizumab-treated patients, and the proportion of MS patients showing levels below the lower limit of normalranged from 5% for IgG and IgA to 30% for IgM (70, 71). Serious infections following episodes of a drop in Ig concentrations below normal limits were rare and most resolved with standard treatment (67).

Infections were reported in 52% of MS patients receiving ofatumumab (mostly nasopharyngitis, upper respiratory and urinary tract infection) and were serious in 2.5% of the cases; herpesvirus associated infections were observed in 4-9% of the patients, being all mild or moderate (69).

The incidence rate of infections was similar between rituximab (68-70%) and placebo-treated (65-71%) patients from the HERMES and OLYMPUS trials, but urinary tract infections and sinusitis were more frequent among rituximab recipients; infection-associated serious adverse events were reported in 3-4.5% of the cases (16, 57).

A recently published Cochrane systematic review reported uncertain about the effect of rituximab on serious adverse events, as these were relatively rare in patients with MS, and they were not well reported in studies; an increased risk of common infections with rituximab was reported, but absolute risk was considered to be small (132).

The incidence of infection was reported to be similar in inebilizumab-treated compared with placebo-treated participants in the N-MOmentum trial, being urinary tract infection (11 vs 9%), more frequent with inebilizumab (38).

*Secondary autoimmunity* was observed following alemtuzumab treatment, and the more frequent events observed were thyroiditis (peak of incidence at year 3: 16%; incidence of serious events <3.5%/year), autoimmune blood and lymphatic system disorders (16-18%, serious in 1-3%; mostly represented by autoimmune thrombocytopenia with annual incidence from 0.1% to 1.1%) and immune-mediated nephropathy (94, 95, 127).

*Malignancies* were reported in 0.5 – 2% of mAbs-treated patients across core studies (60, 69, 70, 127), and no evidence of increased risk compared to demographically matched epidemiological studies was provided by OLE phases (71).

## Future developments

Ab-mediated immune cell depletion is now standard in the therapeutic armamentarium to treat people with MS, and the number and variety of clinically applicable Ab-based cell depleting treatment platforms continues to grow. New developments include approaches that allow Abs to pass the blood brain barrier for more effective depletion of pathogenic immune cells within the central nervous system. The transferrin receptor and the insulin receptor expressed by endothelial cells facilitate receptor-mediated transport through the blood brain barrier and treatment platforms that shuttle immune-cell depleting Abs through these natural brain portals are currently being developed (135, 136).

While rare but serious side effects restricted the use of alemtuzumab, the first immune cell-depleting Ab approved for MS therapy, CD20-targeting B cell-depletion therapies are generally well tolerated and show a favorable safety profile. These therapies were originally developed as a method of eliminating cancerous B cells, and new and emerging cell-depletion approaches in MS have been adopted from protocols used to treat cancer patients. These approaches include targeting of CD19 to affect a wider array of B cells including plasma blasts or CD38, additionally expressed on antibody-producing short- and long-lived plasma cells. Daratumumab, an anti-CD38 mAb initially developed to target tumoral plasma cells in multiple myeloma (137) is also effective in inhibiting clinical activity of antibody-mediated autoimmune diseases, such as autoimmune cytopenia following hematopoietic stem cell transplantation (138) or systemic lupus erythematosus (SLE) (139). The B cell maturation antigen (BCMA) or CD269 is preferentially by mature B lymphocytes, with minimal expression in hematopoietic stem cells or nonhematopoietic tissue, and is essential for the survival of long-lived bone marrow plasma cells, but not overall B-cell homeostasis (140). Anti-BCMA therapies currently being evaluated in clinical trials in patients with multiple myeloma include bispecific Ab constructs, which facilitate cell-to-cell interactions between malignant plasma cells and cytotoxic T cells. Whether plasma cell-depleting therapies show beneficial effects for patients with MS remains to be demonstrated. However, both of the aforementioned molecules, CD38 and BCMA, are attractive targets to deplete long-lived plasma cells in antibody-driven neurological diseases such as NMOSD or antibody-mediated autoimmune encephalitides. T cells genetically engineered to express Ab-based chimeric antigen receptors (CARs) can induce impressive immune responses and achieve remarkable clinical efficacy in patients with certain haematological malignancies. Adoptive transfer of autologous CD19-targeted CAR T cells was among the earliest CAR T cell therapies used in successful clinical trials and the first to gain FDA approval (141). CD19-Targeted CAR T Cells were also reported to induce rapid clinical diseases remission in a patient with difficult to treat SLE (142). The success of Ab-based B cell-depleting therapies in MS suggest that CAR T-cell therapies targeting B-cell antigens should be explored for their potential therapeutic benefit in patients with MS who otherwise have limited treatment options due to high disease activity or poor response to standard treatments. A proportion of MS patients estimated in 4-14% (according to the definition used) can be considered affected by “aggressive MS”, bearing high risk of unfavorable outcomes (143). Although disease activity is controlled by high efficacy DMTs in most of the patients, a not negligible proportion of the cases fails to respond to treatment: as an example, 35% of patients treated with alemtuzumab relapsed by year 2 in the CARE-MS II trial, and 16% of the ocrelizumab-treated patients from the OPERA I-II trials progressed by year 5. The proportion of treatment failure achieves more than 50% of the treated cases when considering NEDA-3 (e.g. 52% for ocrelizumab and 68% for alemtuzumab at year 2) (59, 95). Whether the potential of these new treatment platforms for changing adaptive immune features is superior to currently approved cell-depleting therapies remains to be demonstrated.

During the last two decades the clinical potential of Ab-mediated cell depletion therapy has materialized into therapies that benefit people with MS. With dedicated attention to basic, translational, and clinical research, even more effective, and safe cell-depletion treatment strategies will be developed and likely soon be translated into the clinic.

## Author contributions

AM, PM, and JL contributed to the conception or design of the work, data analysis and interpretation, drafting and critical revision of the article. All authors contributed to the article and approved the submitted version.

## Funding

Work was supported by the German Research Foundation (DFG, SFB-TRR129, to JL)

## Conflict of interest

The handling editor FN declared past co-authorships with the author JL.

The remaining authors declare that the research was conducted in the absence of any commercial or financial relationships that could be construed as a potential conflict of interest.

## Publisher’s note

All claims expressed in this article are solely those of the authors and do not necessarily represent those of their affiliated organizations, or those of the publisher, the editors and the reviewers. Any product that may be evaluated in this article, or claim that may be made by its manufacturer, is not guaranteed or endorsed by the publisher.
